# Stable Extent of Recurrently Active Cardiac and Cutaneous Sarcoidosis

**DOI:** 10.3389/fmed.2021.729229

**Published:** 2021-12-03

**Authors:** Karen C. Patterson, Misha Rosenbach, Paco E. Bravo, Jacob G. Dubroff

**Affiliations:** ^1^Brighton and Sussex Medical School, Brighton, United Kingdom; ^2^Pulmonary, Allergy & Critical Care Division, University of Pennsylvania, Pennsylvania, PA, United States; ^3^Department of Dermatology, University of Pennsylvania, Philadelphia, PA, United States; ^4^Division of Cardiology, Department of Medicine, University of Pennsylvania, Philadelphia, PA, United States; ^5^Division of Nuclear Medicine, Department of Radiology, University of Pennsylvania, Philadelphia, PA, United States

**Keywords:** sarcoidosis, granuloma, cardiac, cutaneous, chronic, recurrence, relapse, inflammation

## Abstract

**Background:** Recurrent or persistently active sarcoidosis is a risk factor for permanent organ damage. Whether this damage is due to accumulated focal injuries or progressive disease extent is not known, as the natural history of chronic inflammation in sarcoidosis is poorly characterized. The objective of this study is to determine the pattern of disease in recurrently active sarcoidosis.

**Methods:** We identified patients with recurrent cardiac sarcoidosis (*N* = 21) retrospectively from an imaging database, and with recurrent cutaneous sarcoidosis (*N* = 17) from a prospective registry. The longitudinal patterns of cardiac sarcoidosis were established by findings on cardiac positron emission tomography scans, and of cutaneous sarcoidosis by the validated Cutaneous Sarcoidosis Activity and Morphology Instrument clinical scoring system. Patterns of recurrent disease were compared to baseline findings.

**Results:** Recurrent sarcoidosis occurred in a nearly identical pattern and distribution as baseline disease, and spread of disease was rarely observed for both cardiac and cutaneous sarcoidosis: 97% of heart segments positive on recurrence scans were positive on baseline scans, and only one new region of facial disease was observed. In some cases, recurrence followed years of apparent remission.

**Discussion:** Across phenotypes, and across a long period of follow-up, the extent of sarcoidosis was stable in spite of fluctuations in disease activity. For patients with a demonstrated history of recurrent disease affecting critical organs, our findings support the need for long-term follow-up.

## Introduction

Sarcoidosis is an inflammatory disease of unknown etiology, marked by the formation of granulomas in affected tissues. The diagnosis is established by compatible clinical and histopathology findings, following the exclusion of other causes of granulomatous inflammation (1). While the lungs and organs of the reticuloendothelial system are the most common sites of disease (2, 3), cardiac involvement affects up to 25% of patients, with an even higher prevalence in Japan (4), and cutaneous involvement also occurs in approximately 25% of cases (5).

Key immunopathologic events in sarcoidosis include an influx of CD4+ T cells, the release of inflammatory cytokines, and the formation of epithelioid granulomas at sites of disease. The oligoclonal expansion of CD4+ T cells and granuloma features support the fundamental assumption that sarcoidosis is antigen driven (6, 7). Yet, the antigenic cause has not been identified, and long-term antigen fate, including whether clearance or development of immune tolerance occurs, is unknown. Disease chronicity is an important prognostic determinant. Nearly all patients who die from sarcoidosis have a chronic form of disease, which is also associated with long-term morbidity (8, 9).

Treatment with immunosuppression can improve symptoms and the function of organs with active sarcoid inflammation. However, for patients with chronically active disease, improvements are often tenuous and several studies have demonstrated a high rate of recurrence following withdrawal of immunosuppression (10–12). Whether disease recurrence leads to a progressive encroachment of normal tissue is important to determine, as the extent of tissue inflammation helps define the risk associated with discontinuation of treatment, particularly for cardiac sarcoidosis where the extent of disease is a critical determinant of mortality (13).

With the hypothesis that the extent of recurrently active sarcoidosis is stable, we serially examined patients with chronic cardiac sarcoidosis and characterized longitudinal disease patterns. To test whether our observations are restricted to a single organ phenotype, we also measured the pattern of recurrently active lymph node disease and, in a separate cohort with cutaneous involvement, of recurrently active cutaneous sarcoidosis. These phenotypes were chosen as they are amenable to anatomical disease tracking via serial assessments of disease activity with [^18^F]fluorodeoxyglucose positron emission tomography (FDG-PET) imaging and expert visual inspection, respectively.

## Materials and Methods

**Figure 1:**
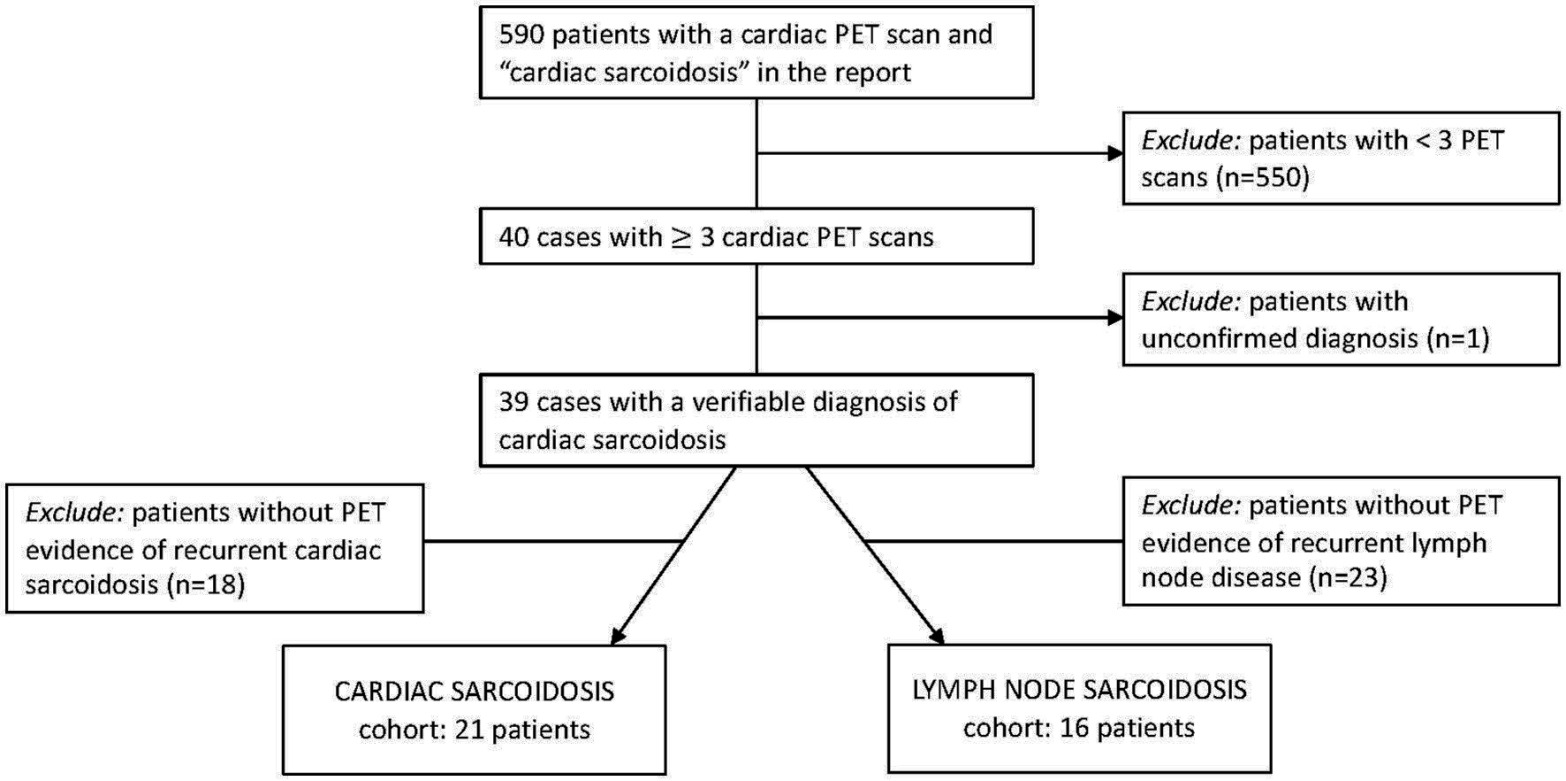
Inclusion criteria to identify patients for two of our final cohorts. Patients from our radiology database were evaluated for PET-determined recurrent cardiac sarcoidosis and recurrent lymph node sarcoidosis.

**Figure 2:**
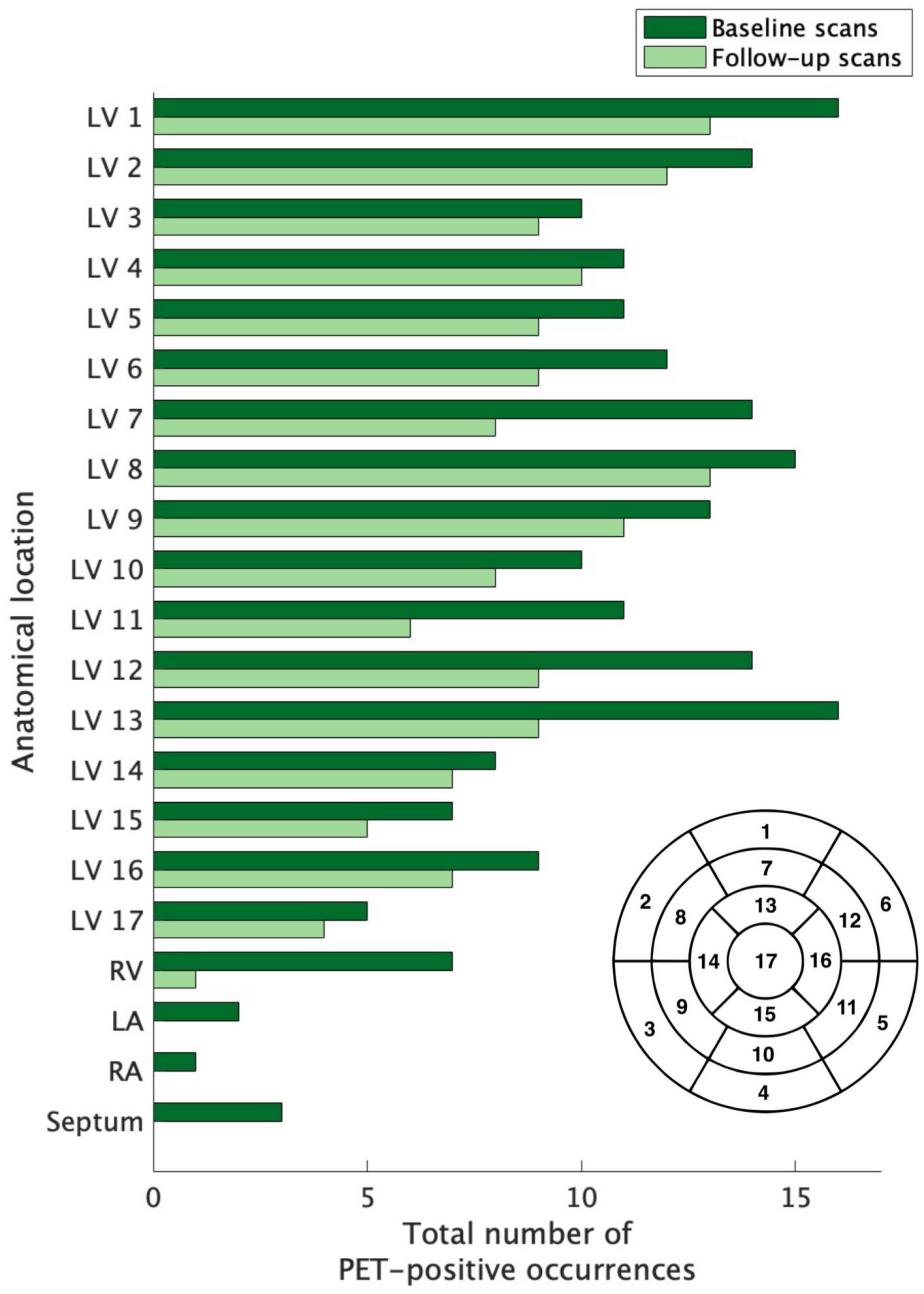
The extent of baseline and recurrent cardiac sarcoidosis. The extent of left ventricle involvement was assessed according to the 17-segment model (lower right of figure) used for grading myocardial damage in ischemic heart disease; the right ventricle, atria, and septum were also evaluated. The extent of active disease for cardiac segments was not equal across compartments. Regions outside of the left ventricle were infrequently affected. Segment 17 was the least commonly affected, but no region of the left ventricle was spared and no region dominated. LV, left ventricle; RV, right ventricle; RA, right atrium; LA, left atrium.

Adult patients seen at the University of Pennsylvania with a diagnosis of sarcoidosis were eligible for this IRB-approved study (protocol numbers 831421, 830093, and 819306). Subjects were accrued from two sources: (1) a clinical database of patients with sarcoidosis who underwent three or more thoracic [^18^F]FDG-PET studies for suspected active myocardial disease from 2006-2018 (Figure 1), and (2) a prospective registry of patients with dermatopathologically verified cutaneous sarcoidosis, enrolled via informed consent. Given the low risk of harm, the need for informed consent for patients with cardiac sarcoidosis was waived by our institutional review board. Demographic data and clinical details were obtained from the electronic medical records. Study data were managed with Research Electronic Data Capture (REDCap) electronic data capture tools (14, 15).

### [^18^F]FDG-PET Evaluations

Depriving normal myocytes of glucose, typically accomplished through a combination of fasting and a low-carbohydrate diet, promotes a metabolic switch to free fatty acid utilization and reduces myocyte uptake of [^18^F]FDG, a glucose analog (16). Under this protocol, enhanced [^18^F]FDG uptake in the heart is presumed to be due to active inflammation, and society guidelines recommend use of [^18^F]FDG-PET for patients with clinical suspicion of myocardial sarcoid involvement (17, 18). Each [^18^F]FDG-PET study, de-identified and consensus-evaluated by two nuclear medicine physicians with expertise in cardiac sarcoidosis, was evaluated for evidence of active myocardial inflammation. For cases with increased FDG uptake in the heart, focal involvement in a pattern typical for cardiac sarcoidosis distinguished true disease from the false-positive condition of incomplete suppression of glucose uptake. Resolution of disease activity was established by the absence of increased radiotracer uptake above blood pool activity. The extent of active disease involving the left ventricle was assessed by identifying the presence or absence of radiotracer using the established according to the American Heart Association 17-segment model (Figure 2).

Baseline and recurrent scans were compared. Where a baseline scan was followed by one or more positive scans, the most extensively affected scan was used for future comparisons. For subjects with recurrent sarcoidosis evident on multiple, sequential scans, we identified the time to first recurrence but, for the comparison to baseline disease, used the most severely affected recurrent scan.

As co-incident thoracic lymph node disease was common among our patients with cardiac sarcoidosis, recurrent lymph node activity was also assessed. The anatomical location of lymph node involvement was determined by assessment of stations which are reliably captured on thoracic PET imaging. In contrast, the lungs were excluded because of known resolution and sensitivity challenges faced by PET.

### Skin Assessments

All adult patients with biopsy proven cutaneous sarcoidosis seen at the University of Pennsylvania Cutaneous Sarcoidosis Clinic are invited to participate in a prospective database study. Patients are seen on average every three months for a routine evaluation and treatment review. Patients undergo a complete skin exam by a single dermatologist (MR) at each visit, with disease activity mapped via the cutaneous sarcoidosis activity and morphology instrument (CSAMI) (19). For this study, the database was queried for patients with at least three visits, who had demonstrated improvement and then subsequent worsening of disease activity consistent with recurrence. Improvement in cutaneous sarcoidosis was defined as a CSAMI score improvement of 5 or more points, which has previously been demonstrated to represent a clinically important difference (20). While all body regions were examined, we limited our analysis to patients with recurrent facial sarcoidosis, as this region was assessed in sub-compartment detail.

### Statistics

Calculations were performed using Graphpad Prism 8.0 (GraphPad Software, Inc., San Diego, CA). Where indicated, data were expressed as means +/-SD.

## Results

From the 39 subjects with cardiac sarcoidosis who had undergone at least three valid cardiac PET scans, 21 had evidence of recurrent disease. Nearly all subjects (18/21) were White, and 71% (15/21) were male (Table 1). The diagnosis of sarcoidosis was established by biopsy in most cases, and all patients with recurrent cardiac sarcoidosis had multi-organ disease (Table 2). From the cutaneous sarcoidosis registry, 17 subjects were identified who had recurrent cutaneous sarcoidosis involving the face. In contrast to cardiac sarcoidosis, the majority of these subjects (14/17) were female and all were Black (Table 1).

**Table 1:** Clinical features of patients with recurrently active sarcoidosis.

	**Cardiac sarcoidosis *N =* 21**	**Cutaneous sarcoidosis *N =* 17**
Age, mean (SD)	52.0 (11.1)	55.4 (9.2)
Sex		
Male, *n* (%)	15 (71%)	3 (12%)
Female, *n* (%)	6 (29%)	14 (82%)
Race		
Black, *n* (%)	3 (14%)	17 (100%)
White, *n* (%)	18 (86%)	0 (0%)

**Table 2:** Sarcoidosis details of patients with recurrently active cardiac sarcoidosis.

**Subject number**	**How diagnosed**	**Organs affected by sarcoidosis**
1	Lung bx	Heart, LN, Lung
2	Kidney and Liver bx	Heart, LN, Lung, Spleen, Skin, Liver, Kidney
3	LN bx	Heart, LN, Lung
4	LN bx	Heart, LN, Lung
5	LN bx	Heart, LN
6	Lung bx	Heart, LN, Spleen
7	Lung bx	Heart, LN, Lung
8	Lung bx	Heart, LN, Lungs, Spleen
9	Clinical presentation/no bx	Heart, LN
10	Cardiac bx	Heart, LN
11	LN bx	Heart, LN, Lung
12	LN bx	Heart, LN, Lung
13	LN bx	Heart, LN, Lung, Bone marrow
14	Clinical presentation/no bx	Heart, LN
15	LN bx	Heart, LN, Lung
16	LN bx	Heart, LN, Lung
17	Lung bx	Heart, LN, Lung
18	Lung bx	Heart, LN, Lung
19	Lung and LN bx	Heart, LN, Lung
20	Eye and LN bx	Heart, LN, Lung, Ocular/CNS
21	LN and heart bx	Heart, LN, Lung

*bx, biopsy; LN, lymph node; CNS, central nervous system*.

### Extent of Cardiac Sarcoidosis at Baseline

Across the 21 subjects with recurrent cardiac sarcoidosis, a combined 163 diagnostic PET scans were available for review. At baseline, cardiac sarcoidosis was highly prevalent throughout the left ventricle (LV). On average, 9.6 segments (56% of all coded segments) on baseline scans demonstrated increased FDG uptake. Certain regions of the LV were more likely to be affected than others (Figure 2). Increased FDG uptake in the right ventricle (RV) was observed in only 33% (7/21) of subjects. Lone RV disease was not observed, and RV involvement was always co-incident with extensive LV disease (range of 11–17 LV segments). In addition, among the 7 subjects with RV involvement, one also had septal involvement, one had septal and left atrium involvement, and one had septal, left atrial, and right atrial involvement.

### Resolution Events in Cardiac Sarcoidosis

**Figure 3:**
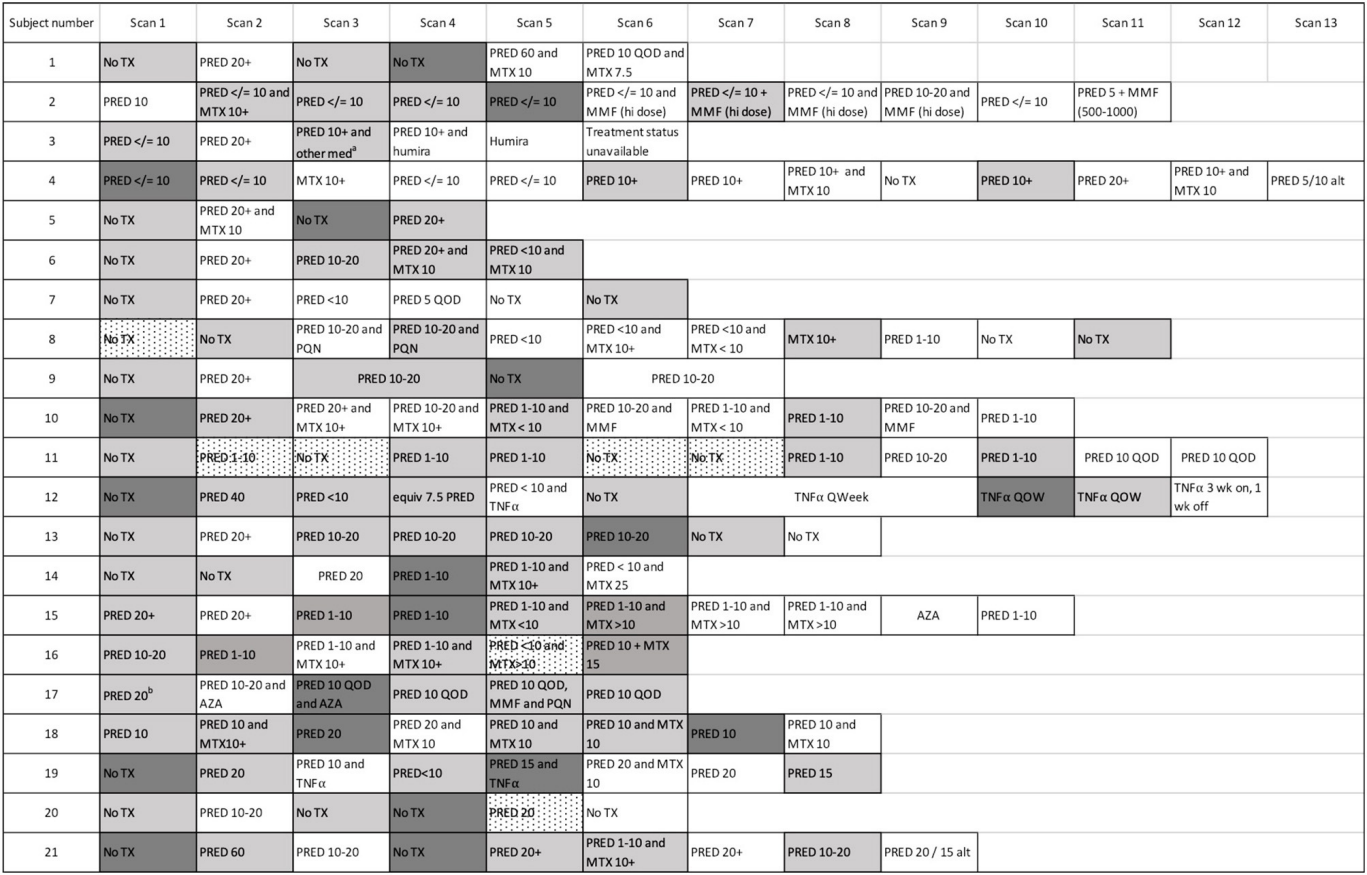
Cardiac PET scan results and associated therapies. The number of scans across the cohort is demonstrated, with the treatment regimen associated with each scan provided. Persistently positive scans (multiple gray blocks in a row) were observed in several cases. “Scan 1” indicates the first available PET scan in our system. ^a^ ‘other med' not qualified in clinical notes. ^b^ Prednisone 20 mg/day had been started several days before this baseline scan. White boxes: negative cardiac PET scan. Light gray boxes: positive cardiac PET scan. Dark gray boxes: scan with most extensive disease, when multiple sequential cardiac PET scans were positive; if serially positive scans demonstrated an equal extent of disease, no dark gray is indicated. Dotted white boxes: non-diagnostic scan due to improper dietary preparation and failed metabolic switching. TX, treatment; PRED, prednisone; MTX, methotrexate; TNFα, tumor necrosis factor-alpha inhibitor; MMF, mycophenolate mofetil; hi, high; AZA, azathioprine; QOD, every other day; Qweek, every week; PQN, plaquenil; alt, alternating days.

The first negative PET scan following a positive baseline or recurrent PET scan defined a resolution event. For over half of these events (25/43, or 58%), resolution was evident on the first scan following the initiation of treatment. However, 18 resolution events followed multiple sequential positive PET scans. For most (16/18) of these events of delayed resolution, the presence of persistently positive PET scans was associated with failure to initiate treatment, or initiation of an insufficient regimen as clearance was eventually achieved following an increase in treatment. Subject 13 (Figure 3), was an exception: their disease appeared to spontaneously remit without treatment. For subject 2, the first two scans were performed in quick succession, and failure to achieve a negative PET scan by the second scan may be due to repeat testing within too short an interval, rather than a true delay in resolution. Indeed, the second scan demonstrated a substantially reduced extent of disease, consistent with an expected response to high dose prednisone, and the third PET scan 8 months later was negative, even as the prednisone dosage had been lowered.

### Recurrent Cardiac Sarcoidosis

**Figure 4:**
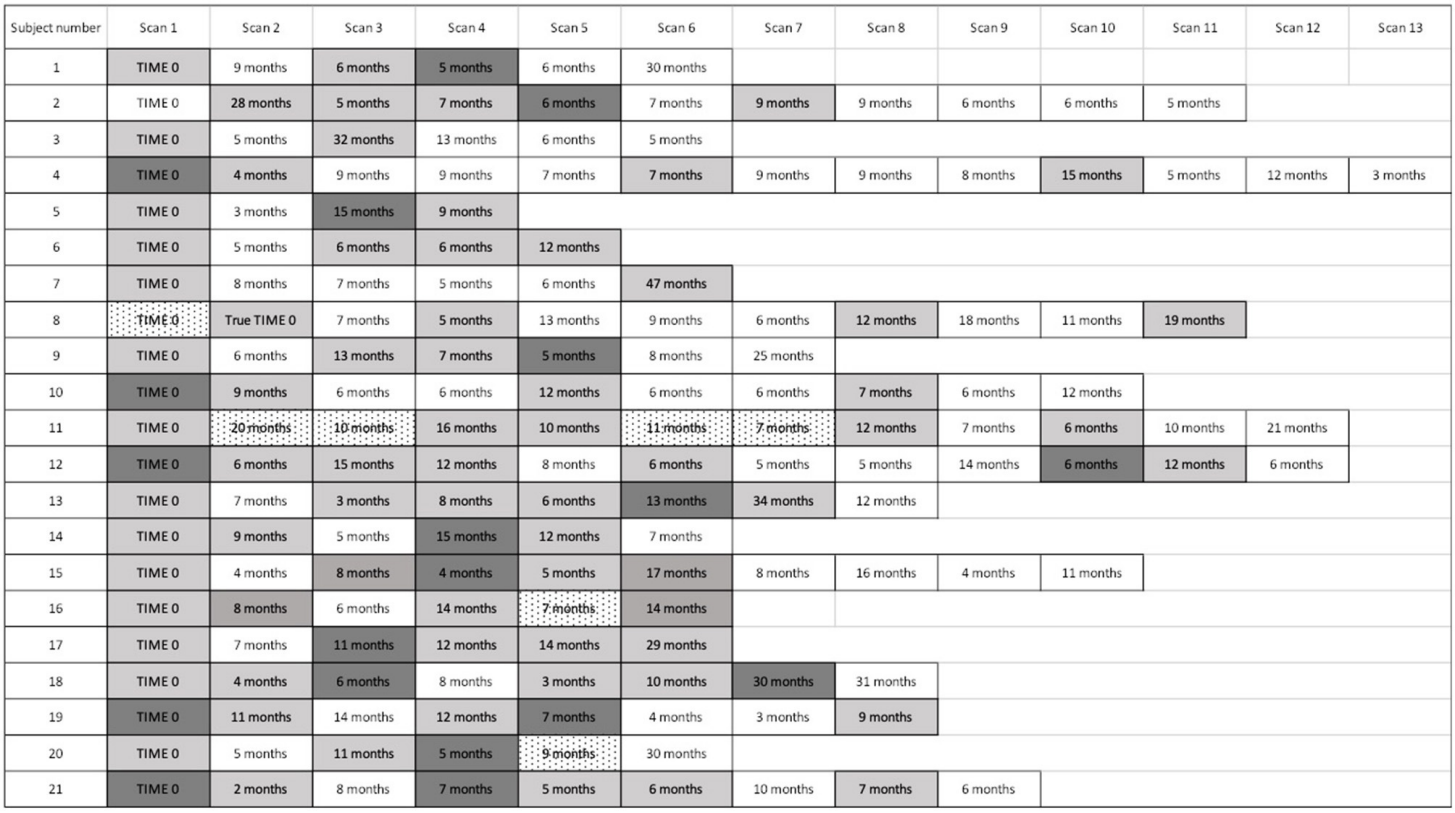
Timeline of cardiac PET scan results. For subjects with recurrently active cardiac sarcoidosis, the number of months from the prior PET scan and PET scan results are displayed. For patients with multiple scans demonstrating disease recurrence, we identified the time to the first sign of recurrence, but used the most severely affected scan to analyse the pattern and extent of recurrence. “Time 0” indicates the first available PET scan in our system. White boxes: negative cardiac PET scan. Light gray boxes: positive cardiac PET scan. Dark gray boxes: scan with most extensive disease, when multiple sequential cardiac PET scans were positive; if serially positive scans demonstrated an equal extent of disease, no dark gray is indicated. Dotted white boxes: non-diagnostic scan due to improper dietary preparation and failed metabolic switching.

Figure 4 displays the timeline of scans for each subject. The average time between baseline and final PET scan, representing the total length of follow-up, was 5.8 years (SD 2.1, range 2.2 to 10.7 years). During this time, 28 episodes of recurrent cardiac sarcoidosis occurred among the 21 subjects. While most had a single recurrence, five subjects had two recurrence episodes, and one subject had three recurrence episodes. In nearly all cases, recurrence followed a reduction or cessation in treatment (Figure 3).

**Figure 5:**
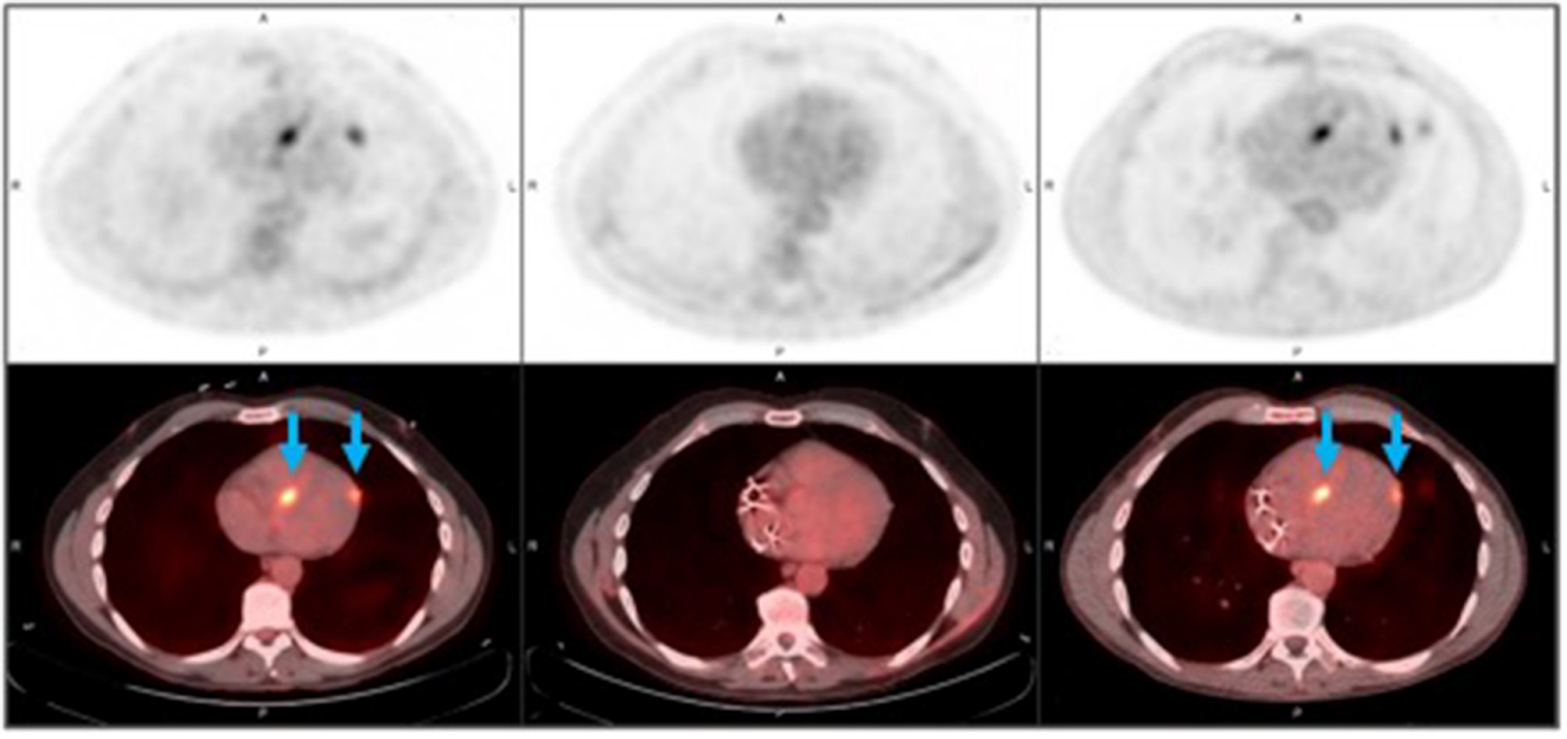
Recurrent cardiac sarcoidosis. These [^18^F]FDG-PET/CT axial images observed in a 53 year-old male represent findings in recurrent cardiac sarcoidosis. The top row demonstrates FDG-PET images, and the bottom row demonstrates FDG-PET images fused with CT imaging. Foci of increased FDG uptake (blue arrows) are representative findings in recurrent cardiac sarcoidosis present in the mid septal and mid lateral walls (first column), then resolve with therapy (middle column), and then recur in the same location (third column). By column, images are displayed chronologically. The left column captures pre-treatment disease features; these can be compared to imaging features of disease recurrence depicted in the right column (performed 10 months after the initial, pre-treatment scan). Images in the middle column negative scan (performed 5 months after the initial, pre-treatment scan) demonstrate the disappearance of metabolically-manifest disease activity.

For the majority of subjects (17/21), the distribution of recurrent sarcoid inflammation occurred at anatomical locations that were grossly identical to the location of original lesions (Figure 5). Of the 175 segments positive among the recurrence scans across all subjects, 169 (97%) were positive on baseline scans. For four subjects, disease recurrence occurred in a similar distribution but newly positive segments were also evident. One of these subjects was on treatment at the time of the baseline scan, and thus the appearance of newly positive regions is possibly an artifact related to an incomplete baseline assessment. Among the other three, 23/28 (82%) of the segments positive for FDG uptake on recurrence scans were positive on baseline scans, with an additional five segments newly positive. Only one subject experienced recurrent RV involvement; this was concomitant with recurrent LV disease, and the location of recurrence was the same as baseline disease. While recurrent cardiac disease nearly always occurred in locations of previously active disease, the average extent of recurrent disease (42% of all coded segments were positive) was lower than that of baseline disease (56% of all coded segments were positive).

### Recurrent Cutaneous Sarcoidosis

**Figure 6:**
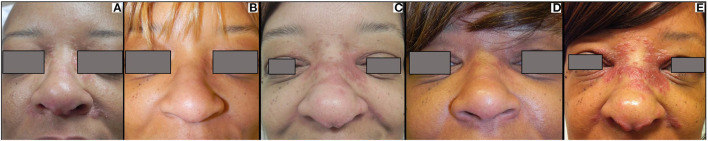
Recurrent cutaneous sarcoidosis. This patient has extensive papular sarcoidosis of the medial canthus, nose, and melo-labial crease **(A)**. Her disease initially cleared with hydroxychloroquine and minocycline therapy **(B)**. She experienced disease recurrence **(C)**, and after failing to respond to methotrexate, was treated with infliximab with clearance **(D)**. When her infliximab was stopped, she exhibited recurrence in a similar distribution with minimal local direct extension **(E)**. She has since cleared again on infliximab (not shown).

Of the 104 patients in the cutaneous sarcoidosis database, 38 had three or more visits. Of those, 17 had a CSAMI score decrease of >5 at a follow-up visit, followed by recurrent disease involving the face. Facial sub-compartments assessed on serial skin exams included the nose, cheeks, perioral regions, and ears. Patients had on average 2 regions affected by sarcoidosis. Peri-nasal disease (14/17 patients at baseline, 13 of whom had recurrence at this site) was most common, followed by disease involving the cheek (13/17 patients at baseline, 12 of whom had recurrence at this site). Perioral disease was observed in 9/17 subjects at baseline, 7 of whom had disease recurrence at this site. Ear involvement was less common (3/17 at baseline). For 94% (16/17) of cases, recurrently active lesions occurred in the same region as initial disease activity (Figure 6). Direct local extension was observed occasionally, although the overall extent of disease was deemed non-progressive based on examination documentation and photographic tracking. For one case, disease recurrence was similar to baseline disease with one new region also affected. Similar to cardiac sarcoidosis, the average extent of recurrent disease (34 facial regions among 17 patients) was lower than baseline disease (39 regions among 17 patients).

### Recurrent Lymph Node Sarcoidosis

A total of 16 subjects had PET scan findings consistent with recurrent lymph node inflammation; 12 were from the recurrent cardiac sarcoidosis cohort and an additional four subjects were identified from the 37 subjects with cardiac sarcoidosis screened for recurrent disease (Figure 1B). While recurrent lymph node disease tended to occur in a similar distribution to baseline disease (Supplementary Figure 1), this pattern was less consistent than that observed for cardiac and cutaneous sarcoidosis. Across the 16 patients, 82 lymph node stations had increased FDG uptake on recurrence scans, 24 (29%) of which were newly positive compared to baseline scans.

## Discussion

We found that recurrent cardiac sarcoidosis has a strong predilection to recur in a distribution similar to previously active disease. The same finding was observed for patients with recurrently active cutaneous sarcoidosis. The finding that the extent of disease remains remarkably stable over time was observed over many years of follow-up. Our data suggest that, for a given organ affected by sarcoidosis, persistent or recurrent inflammation, with the potential transition to irreversible fibrosis, are more concerning risks than local expansion of disease. For approximately half of our cardiac sarcoidosis cohort, recurrence events either did not trigger initiation of treatment or were followed by an insufficient regimen such that multiple serial cardiac PET scans were positive, in some cases resulting in active disease spanning several years. For these cases, disease extent still remained stable. Therefore, even in the setting of persistently active inflammation, cardiac sarcoidosis does not appear to have a high propensity to progress beyond the initial extent of disease. This novel finding has profound implications for understanding and treating chronic sarcoidosis.

Lymph node involvement is common in sarcoidosis, and many of our patients had their diagnosis established by lymph node biopsy. However, the specificity of PET imaging for nodal sarcoidosis activity is unknown, and increased nodal FDG uptake may be seen in a variety of inflammatory conditions. In contrast to the findings for cardiac and cutaneous sarcoidosis, the distribution of thoracic lymph node activity was more variable over time, with a higher rate of new FDG avid lymph node stations observed on recurrently positive PET scans. This finding may be due to a lower specificity of PET imaging for nodal sarcoidosis activity, with the possibility of false positive findings on follow-up scans. Alternatively, the lymph node network truly may be more prone to a progressive extent of disease in patients with chronic sarcoidosis.

The stability of lesional boundaries in cardiac and cutaneous sarcoidosis contrasts with the possibility that the systemic burden of sarcoidosis may increase over time, which has been observed in previous studies of patients followed longitudinally (21). An increase in the total body burden of disease is a clinically important aspect of ‘progressive sarcoidosis'. We acknowledge that the stable extent of local disease observed in our cohort does not predict or protect against systemically progressive disease.

While immunosuppression therapy is often successful in suppressing inflammation, disease recurrence following cessation is a vexing outcome in both cardiac and cutaneous sarcoidosis (22). The optimal duration of treatment for active sarcoidosis remains unclear (23). For patients with potentially dangerous disease, a prolonged course is often undertaken, guided by hope that sustained control of inflammation will reduce the risk of recurrence (8). Yet, the ability of a prolonged treatment course to reliably achieve lasting remission has been questioned (24). In our cohort, we observed the recurrence of cardiac sarcoidosis following treatment courses that approached or exceeded two years. This raises concern that, at least among patients with a demonstrated propensity for recurrent disease, long-term remission off treatment is not readily predicted by a lengthy treatment course.

The mechanism of recurrent sarcoidosis is unknown. In a study on recurrent Lofgren's disease, repeat exposure to antigen was invoked to account for episodes of recurrence occurring remote from the time of initial disease (25). In contrast, based on our findings we speculate that failure to clear antigen may contribute to chronic sarcoidosis, as the recrudescence of inflammatory lesions in a stable distribution suggests that antigen may not be cleared between periods of active disease. Rather, the inflammatory response to antigen may be “temporarily compromised” (26) during periods of disease quiescence. This quiescence can be fragile. While further work is needed to explore the long-term stability of remission in patients with previously recurrent sarcoidosis, sustained antigen tolerance is a plausible mechanism. In contrast to tolerance, findings from a Phase I preclinical sarcoidosis trial suggested that enhancing host immune responses may facilitate antigen clearance, lending hope for true long-term disease remission (27).

One cautious interpretation of our findings is that the containment of antigen is not compromised over time. Altered immunity has been implicated in antigenic spread in tuberculosis, another granulomatous disease (28, 29). Yet, the stable macroscopic extent of inflammation observed in our cohort suggests that antigen continues to be well-contained within original areas of granulomatous lesions, along with the potential persistence of resident memory immune cells.

There are several important limitations of this study. The ability to anatomically localize inflammatory lesions by imaging techniques, such as PET, is inherently imprecise. We cannot rule out that minor or microscopic changes in the extent of disease occurred. We found that the average extent of disease was lower at disease recurrence compared to baseline scans. However, treatment was often started or increased upon disease recurrence, which may chemically constrain the extent of inflammation. This single site study has a modest cohort size; larger studies are needed to more robustly establish the patterns of chronically active disease. For two subjects with cardiac sarcoidosis, biopsy was deferred after a careful risk-benefit assessment, and the diagnosis was established on other clinical grounds. While a common situation, the diagnosis is less certain in such cases. Finally, we recognize the stark differences in demographics between our cohorts of cardiac and cutaneous sarcoidosis. These differences raise concern that identification of cardiac sarcoidosis, which is often done through screening studies initiated by health care providers, may be influenced by demographics. Differences in access to health care may also play a role.

We focused on the evaluation of organs which tend to have countable and localizable lesions, and our findings may not be generalizable to all phenotypes of sarcoidosis. An especially important question un-answered by our study is the nature of recurrent lung sarcoidosis. Tracking individual lung lesions, which are often tiny, clustered, or indistinct, is inherently less precise than tracking the more distinct cardiac and cutaneous lesions. Yet, a better understanding of how pulmonary sarcoidosis progresses is critically important as chronic lung disease, along with cardiac involvement, accounts for the majority of fatal outcomes in sarcoidosis (30). While we focused on the fate of single organs affected by sarcoidosis, we acknowledge that sarcoidosis is a multi-system disease, and indeed nearly all of our subjects had documented involvement of at least two organ systems. Future studies to assess the course of multi-organ disease will be important to determine how closely disease activity in one organ compartment relates to disease activity in another.

In conclusion, the evolution of sarcoidosis has been a key unknown of the disease process, and a challenge for clinical management. In our cohorts of cardiac and cutaneous sarcoidosis, the extent of inflammation in recurrently active disease was strikingly stable. The anatomical fidelity of recurrent disease activity raises the possibility that the sarcoid antigen is contained but not cleared. A paradigm shift away from sole reliance on long-term immunosuppression to the development of therapeutics which target immune clearance or halt fibrotic transformation is needed. Our findings also indicate that, for patients with demonstrated disease recurrence or with high-stakes organ involvement, long-term follow-up is warranted even for those who appear to be in clinical remission soon after stopping treatment.

## Data Availability Statement

Upon request, the raw data supporting the conclusions of this article will be available by the authors.

## Ethics Statement

The studies involving human participants were reviewed and approved by University of Pennsylvania Institutional Review Board. Written informed consent for participation was obtained for patients in the cutaneous sarcoidosis cohort, but was not required for the cardiac sarcoidosis cohort in accordance with institutional requirements. Written informed consent was obtained from the individual(s) for the publication of any potentially identifiable images included in this article.

## Author Contributions

KP had full access to all of the data in the study and takes responsibility for the integrity of the data and the accuracy of the data analysis. All authors have made substantial contributions to the work reported in this manuscript, including study design, data collection and analysis, and writing and editing. All authors contributed to the article and approved the submitted version.

## Conflict of Interest

The authors declare that the research was conducted in the absence of any commercial or financial relationships that could be construed as a potential conflict of interest.

## Publisher's Note

All claims expressed in this article are solely those of the authors and do not necessarily represent those of their affiliated organizations, or those of the publisher, the editors and the reviewers. Any product that may be evaluated in this article, or claim that may be made by its manufacturer, is not guaranteed or endorsed by the publisher.

## Supplementary Material

Supplementary Figure 1 The extent of baseline and recurrent thoracic lymph node involvement. The hilar, subcarinal, and right upper paratracheal lymph nodes were the most frequently affected, with no cases pre-vertebral or pericardial lymph nodes with increased FDG uptake. Overall, similar to cardiac sarcoidosis, there were fewer positive findings for lymph nodes at follow-up compared to baseline scanning. Rt, right; Lt, left; paratrach, paratracheal node; Prevasc, prevascular node; Prevert, prevertebral node; AP, Aortopulmonary; Paraesophag, Paraesophageal node; Pulm lig, pulmonary ligament node; Ant diaphragm, anterior diaphragmatic node; Pericard, pericardial node; supraclav, supraclavicular node; axill, axillary node.

## Abbreviations

IRB: Institutional Review Board
FDG-PET: [^18^F]fluorodeoxyglucose positron emission tomography
REDCap: Research Electronic Data Capture
CSAMI: cutaneous sarcoidosis activity and morphology instrument
LV: left ventricle
RV: right ventricle
MRI: magnetic resonance imaging
EF: ejection fraction.
