# Supplementary figures and images for: Stable Extent of Recurrently Active Cardiac and Cutaneous Sarcoidosis
Source: Front Med (Lausanne). 2021 Dec 3;8:729229. doi: 10.3389/fmed.2021.729229 (PMC8677932; doi:10.3389/fmed.2021.729229)

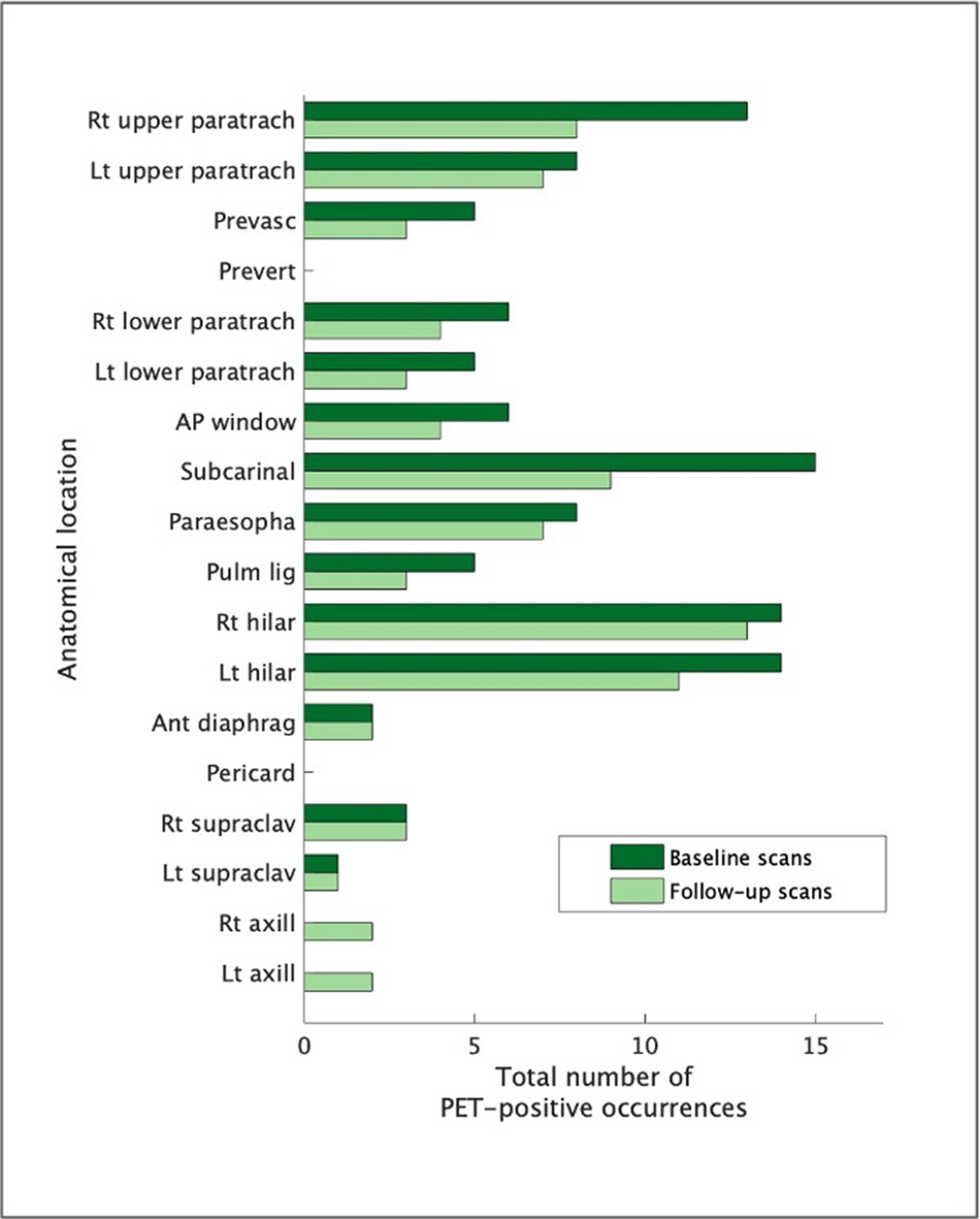

Supplement: Supplementary Figure 1 — The extent of baseline and recurrent thoracic lymph node involvement. The hilar, subcarinal, and right upper paratracheal lymph nodes were the most frequently affected, with no cases pre-vertebral or pericardial lymph nodes with increased FDG uptake. Overall, similar to cardiac sarcoidosis, there were fewer positive findings for lymph nodes at follow-up compared to baseline scanning. Rt, right; Lt, left; paratrach, paratracheal node; Prevasc, prevascular node; Prevert, prevertebral node; AP, Aortopulmonary; Paraesophag, Paraesophageal node; Pulm lig, pulmonary ligament node; Ant diaphragm, anterior diaphragmatic node; Pericard, pericardial node; supraclav, supraclavicular node; axill, axillary node. [file Image_1.JPEG]
